# The BisPCR^2^ method for targeted bisulfite sequencing

**DOI:** 10.1186/s13072-015-0020-x

**Published:** 2015-08-01

**Authors:** Diana L Bernstein, Vasumathi Kameswaran, John E Le Lay, Karyn L Sheaffer, Klaus H Kaestner

**Affiliations:** Department of Genetics and Institute for Diabetes, Obesity, and Metabolism, Perelman School of Medicine, University of Pennsylvania, 3400 Civic Center Blvd., Philadelphia, PA 19104 USA

**Keywords:** Targeted bisulfite sequencing, DNA methylation, Next-generation sequencing

## Abstract

**Background:**

DNA methylation has emerged as an important regulator of development and disease, necessitating the design of more efficient and cost-effective methods for detecting and quantifying this epigenetic modification. Next-generation sequencing (NGS) techniques offer single base resolution of CpG methylation levels with high statistical significance, but are also high cost if performed genome-wide. Here, we describe a simplified targeted bisulfite sequencing approach in which DNA sequencing libraries are prepared following sodium bisulfite conversion and two rounds of PCR for target enrichment and sample barcoding, termed BisPCR^2^.

**Results:**

We have applied the BisPCR^2^ technique to validate differential methylation at several type 2 diabetes risk loci identified in genome-wide studies of human islets. We confirmed some previous findings while not others, in addition to identifying novel differentially methylated CpGs at these genes of interest, due to the much higher depth of sequencing coverage in BisPCR^2^ compared to prior array-based approaches.

**Conclusion:**

This study presents a robust, efficient, and cost-effective technique for targeted bisulfite NGS, and illustrates its utility by reanalysis of prior findings from genome-wide studies.

**Electronic supplementary material:**

The online version of this article (doi:10.1186/s13072-015-0020-x) contains supplementary material, which is available to authorized users.

## Background

DNA methylation refers to the addition of a methyl group to the 5-carbon position of cytosine residues, and in mammalian genomes occurs most commonly in the context of CpG dinucleotides. As an epigenetic mark, this chemical modification does not alter the DNA sequence, but rather regulates transcriptional programs to direct processes such as cellular differentiation, genomic imprinting, and X-chromosome inactivation, while promoting genomic stability [1–4]. The majority of CpGs throughout the mammalian genome are fully methylated, while the remainder exists in an unmethylated or lowly methylated state, corresponding to active regulatory elements such as promoters and enhancers [4–8]. Aberrant DNA methylation has been implicated in an increasing number of morbidities, particularly cancer and aging-associated diseases such as type 2 diabetes, neurological disorders, and cardiovascular disease [9–11]. Many of the studies linking DNA methylation to disease have been prompted by the observation that only a small fraction of the inherited risk of these complex disorders can be explained by genetic variation, as determined by genome-wide association studies (GWAS) [12, 13]. DNA methylation, along with other epigenetic alterations, may provide the link between environmental factors or intrauterine exposure and complex disorders.

A key challenge in the epigenetics field has been achieving high-resolution genome-wide detection of these modifications in sufficient sample sizes to make claims about disease association. In mapping DNA methylation, the most advanced technologies include array-based techniques such as the Infinium HumanMethylation450 BeadChip, which assays 450,000 individual CpGs among 99% of RefSeq genes, and whole genome shotgun bisulfite sequencing (WGBS), which maps cytosine methylation across the entire genome at single base resolution, covering approximately 30 million CpGs. While array-based approaches are more cost-effective and higher throughput, the restrictive sampling of CpGs provides an incomplete landscape of the methylome [14]. However, WGBS experiments are extremely resource-intensive, because exhaustive sequencing is required to achieve sufficient coverage to accurately determine the percentage of methylation at all CpGs. Therefore, it is only practical to conduct WGBS on a limited number of samples, and coverage is usually in the range of 5–15X per CpG, limiting statistical significance of findings. In both instances, novel findings need to be validated in larger populations through targeted methylation analyses. Thus, there is an increasing need for targeted sequencing techniques that are high-throughput, cost-effective, and provide single base resolution.

Next-generation sequencing (NGS) strategies have been developed as an alternative to fluorescence-based pyrosequencing, which is limited by the number of samples that can be processed, and the fact that its short read lengths cover only a few CpGs at a time. These protocols entail PCR amplification of target regions from bisulfite-converted genomic DNA, followed by DNA sequencing library preparation using techniques such as standard Illumina protocols or transposase-based Nextera XT technology [15, 16]. While providing precise and accurate DNA methylation data with high statistical significance, DNA sequencing library preparation is quite expensive and cumbersome when evaluating large numbers of samples or target regions.

**Fig. 1:**
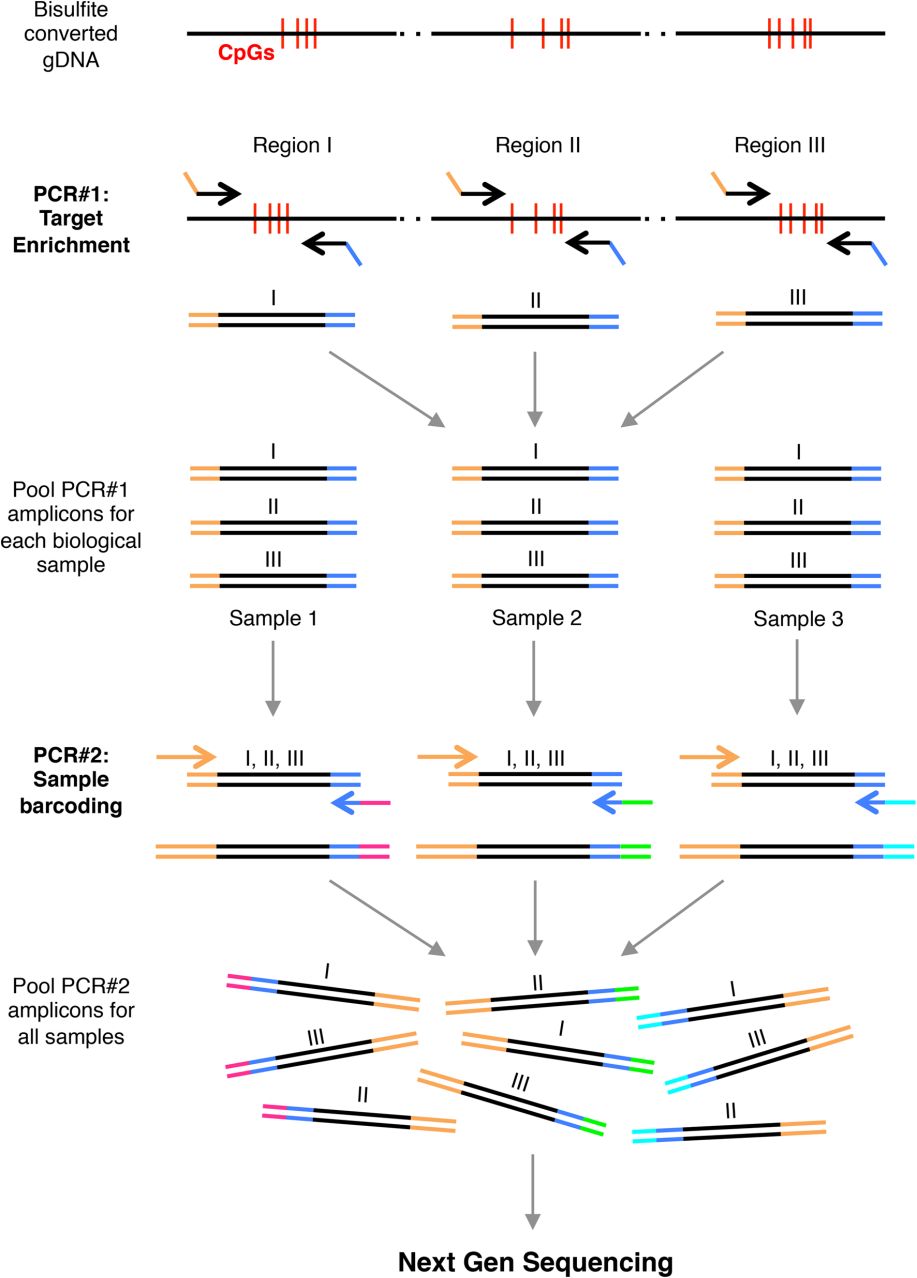
Schema of BisPCR^2^ method for targeted bisulfite sequencing. DNA sequencing libraries are prepared by bisulfite conversion of genomic DNA followed by two rounds of PCR for target enrichment (PCR#1) and subsequent sample barcoding (PCR#2). Partial adapter overhangs are added to target enrichment primers to permit simplified library preparation by PCR. PCR#1 amplicons are pooled prior to the PCR#2 reaction for each biological sample. Due to the presence of the unique barcodes, all PCR#2 amplicons can be pooled for a single next-generation sequencing run.

Therefore, we have developed a novel approach for constructing targeted bisulfite NGS libraries that are prepared by bisulfite conversion of genomic DNA followed by two rounds of PCR, termed BisPCR^2^, eliminating the need for traditional DNA library preparation procedures (Fig. 1). In the BisPCR^2^ method, the entire library preparation process has been reduced to a single 50-min PCR reaction. We have validated the usefulness of this method in the context of type 2 diabetes, first confirming reported differences in DNA methylation at the imprinted *MEG3* locus, and by validation of previous genome-wide findings of CpG risk loci identified in type 2 diabetic human islets [17, 18].

## Results

### The BisPCR^2^ method for targeted bisulfite sequencing

In order to simplify targeted bisulfite NGS, we developed a PCR-based method for library preparation, termed BisPCR^2^ (Fig. 1). The first step in this procedure is sodium bisulfite treatment of genomic DNA (gDNA), which deaminates unmethylated cytosines to uracils, while methylated cytosines are protected. In subsequent PCR amplification reactions, uracils are amplified and ultimately sequenced as thymine residues. The goal of the first PCR (PCR#1) is target enrichment to amplify regions of interest from bisulfite-converted gDNA. The target enrichment primers have overhangs with partial adapter sequences that are subsequently used to amplify barcoded libraries in the second round of PCR (PCR#2). Target enrichment PCR products (PCR#1) for each sample are pooled prior to PCR#2 to simultaneously add the same multiplexing indices to all amplicons of interest. A detailed diagram of BisPCR^2^ primer design is provided in Additional file 1: Figure S1. Following sample barcoding, all PCR#2 reactions are purified and pooled for sequencing on the Illumina Miseq with 150 base pair paired-end reads. We found that purification of final libraries with AMPure XP beads efficiently removed primer dimers in comparison to column based PCR purification (data not shown). To prove that BisPCR^2^ is comparable to traditional targeted bisulfite NGS approaches, we measured DNA methylation at the H19 locus in mouse genomic DNA using both methods and found nearly identical results (Additional file 2: Figure S2).

### BisPCR^2^ library construction and sequencing

**Fig. 2:**
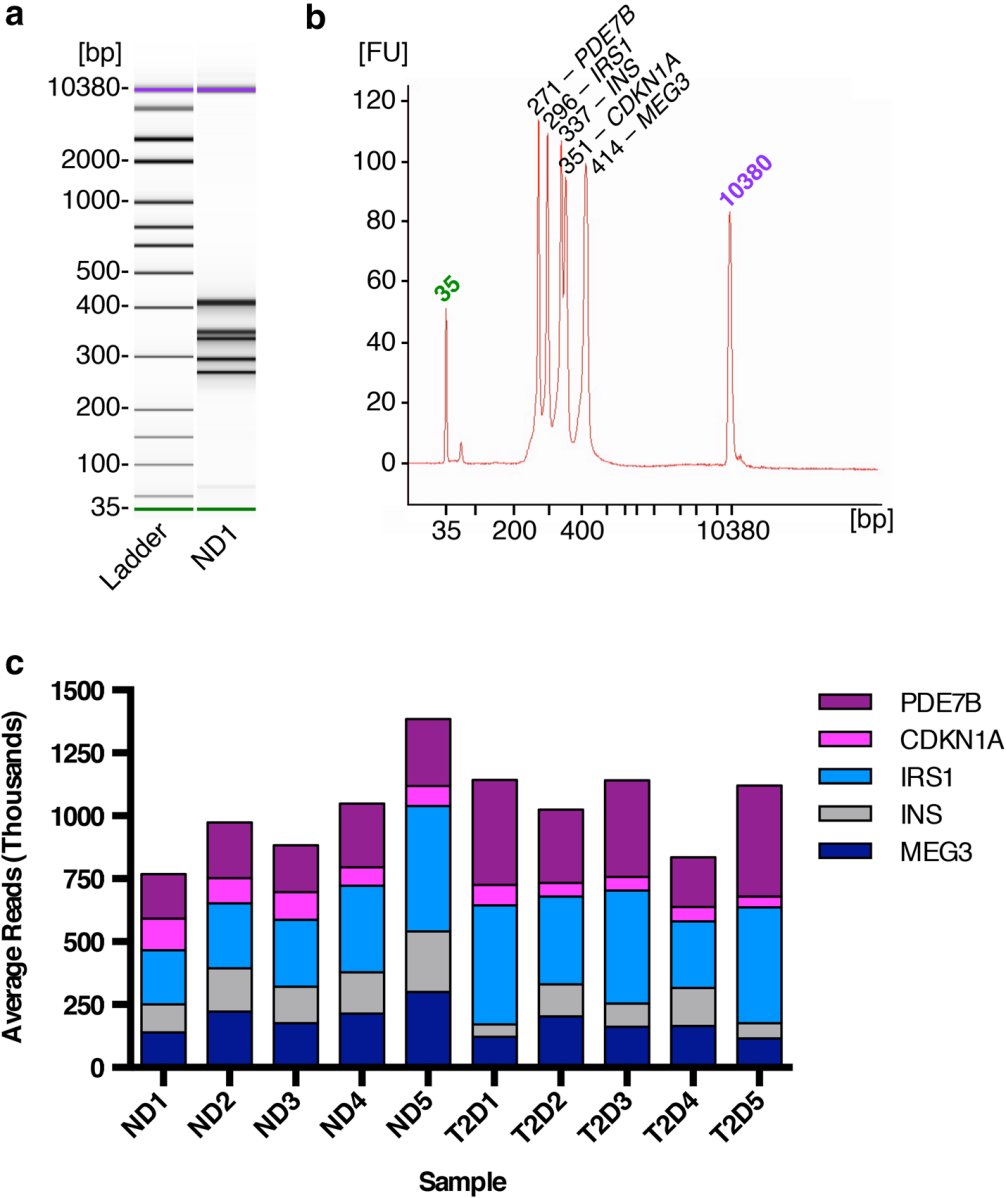
BisPCR^2^ DNA sequencing libraries. **a** Bioanalyzer gel visualizing the five amplicon fragments of a representative sample, ND1, following PCR#2. **b** Bioanalyzer electropherogram quantifying the amount of each fragment in ND1, illustrating the roughly equivalent amounts of all five amplicons. **c** Average reads per amplicon for each sample. *ND* non-diabetic, *T2D* type 2 diabetic.

In this study, we selected five target loci for evaluation, as described below, and compared their DNA methylation profile in five non-diabetic and five type 2 diabetic human islet samples. Pancreatic islet donor information is provided in Table 1. Thus, for each of these ten biological samples, five PCR#1 amplicons were pooled, purified, and then used as template for the PCR#2 barcoding reaction. Target regions ranged in size from 171 to 298 bps (Table 2), and PCR#2 conditions were optimized to prevent amplification bias, particularly of smaller fragments, with the goal of balancing each library with roughly equivalent amounts of each amplicon (Fig. 2a, b).

**Table 1:** Human pancreatic islet donor information

Donor	Gender	Age (years)	BMI (kg/m^2^)
Non-diabetic 1	M	50	29.1
Non-diabetic 2	F	59	28.3
Non-diabetic 3	M	49	31.3
Non-diabetic 4	M	60	22.5
Non-diabetic 5	M	51	38.9
Type 2 diabetic 1	M	58	29.3
Type 2 diabetic 2	M	43	37
Type 2 diabetic 3	F	40	33.9
Type 2 diabetic 4	F	57	48.4
Type 2 diabetic 5	M	47	NA

**Table 2:** Description of PCR products assessed for DNA methylation analysis

Locus	Coordinates	Region length (bp)	Final amplicon (bp)
MEG3	chr14: 101,291,952-101,292,257	298	420
INS	chr11: 2,182,551-2,182,775	225	347
IRS1	chr2: 227,659,611-227,659,781	171	293
CDKN1A	chr6: 36,645,462-36,645,696	235	357
PDE7B	chr6: 136,172,765-136,172,917	153	275

The MiSeq sequencing run produced 14.15 million reads, with 12.75 million passing filter. The sample was spiked with 10% PhiX control, and 8.5% of total reads were aligned to the PhiX genome. Approximately 85% of remaining reads, or 10 million, were aligned to the human genome. Therefore, the expected number of reads per amplicon per sample was approximately 200,000 reads. The percentage of reads allocated to each of the ten samples ranged from 7.01 to 12.45% (Fig. 2c). The slight deviation from the expected 10% per sample is likely due to small pipetting errors when preparing the sequencing pool. Across all samples, the average read number per locus was 206,411, ranging from 78,000 to 358,000 reads (Fig. 2c). The range in sequencing depth is likely due to imprecise pooling of PCR#1 products. The amount of each PCR#1 product pooled was based on relative band intensity of one representative sample, non-diabetic 1 (ND1), run on a 1.5% agarose gel, and does not account for sample to sample variability, which we anticipated to be low. This approximation is suitable for many applications of the BisPCR^2^ method, although samples could be assessed for pooling independently if so desired. Nevertheless, even the minimal read depth of 78,000 allows for exceedingly precise determination of methylation levels.

### Validation of type 2 diabetes differentially methylated loci

We tested our BisPCR^2^ targeted bisulfite sequencing approach by measuring DNA methylation of the promoter region of *MEG3* in human pancreatic islets. *MEG3* is a complex imprinted locus that produces 54 microRNAs, the *MEG3* lncRNA, and multiple additional small RNA species. *MEG3* was shown to be down-regulated with corresponding promoter hypermethylation in type 2 diabetic (T2D) human islets [17]. As an imprinted locus, the *MEG3* promoter is expected to be approximately 50% methylated in normal human islets, and thus it is an ideal target for validating the BisPCR^2^ strategy.

**Fig. 3:**
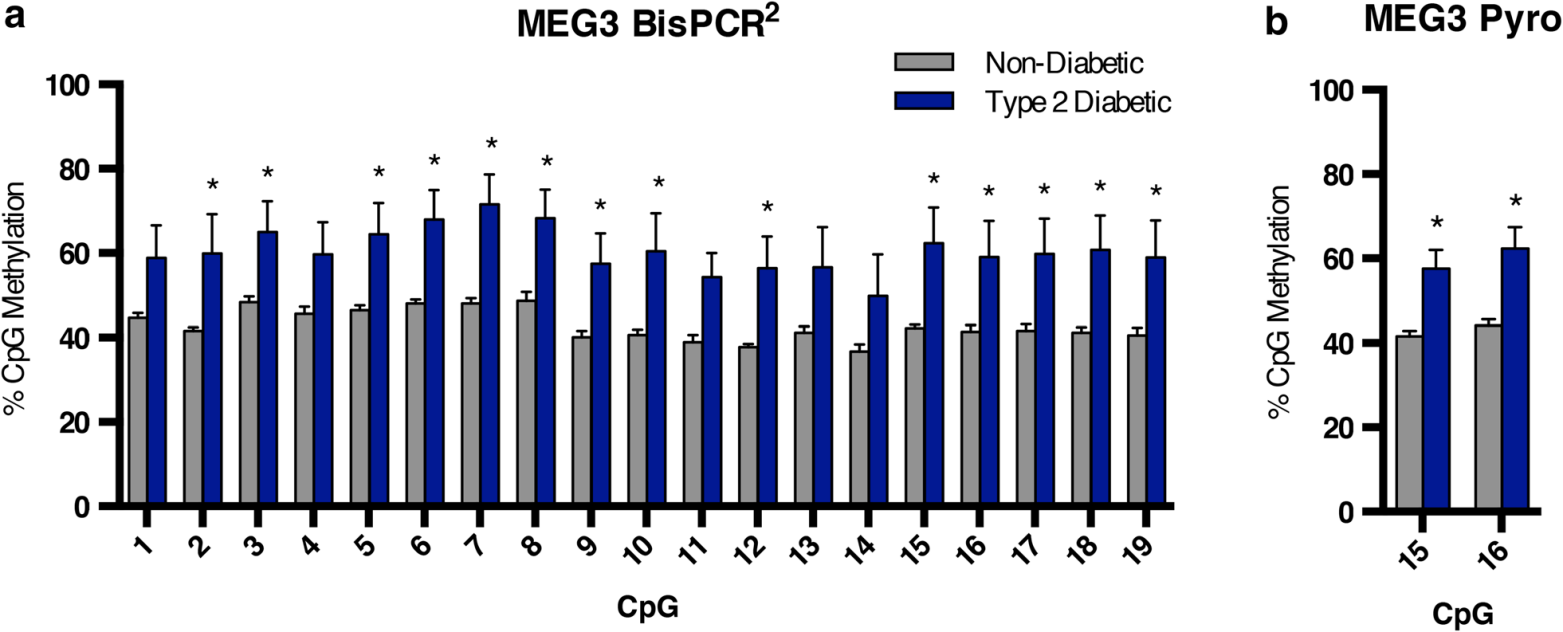
BisPCR^2^ DNA methylation analysis confirms increased CpG methylation in type 2 diabetic human islets at the *MEG3* locus. **a** Average percent CpG methylation at the *MEG3* locus for five non-diabetic and five type 2 diabetic human islet samples measured by BisPCR^2^. *p* values calculated by a two-tailed *t* test. *p < 0.05. *Error bars* indicate SEM. **b** Quantification of average percent CpG methylation by pyrosequencing using the same samples and same *MEG3* PCR primer sequences as in **a**. Only 2 of 19 CpGs are covered in the pyrosequencing assay. Data displayed as in **a.**

Target enrichment primers were designed to amplify a 298 base pair region within the *MEG3* promoter at position −188 to −493 relative to the transcription start site. This amplicon covered 19 CpGs, and the average CpG methylation across the region was significantly increased from 43% in non-diabetic to 61% in type 2 diabetic human islets (p < 0.0001), confirming the report by Kameswaran and colleagues. Of the 19 CpGs covered, 14 had significantly increased CpG methylation in type 2 diabetics (p < 0.05) (Fig. 3a). To further corroborate our findings, using primers directed to the same target region, we technically validated our results with pyrosequencing. Although the same target region was analyzed, the fluorescence-based pyrosequencing reaction covered only 2 of the 19 CpGs within the amplicon, #15 and #16. These 2 CpGs showed comparable levels of CpG methylation in the non-diabetic and type 2 diabetic samples as the BisPCR^2^ method (Fig. 3b). Thus, we were able to technically validate our results with pyrosequencing, and analyze ten times as many CpGs with the BisPCR^2^ method.

**Fig. 4:**
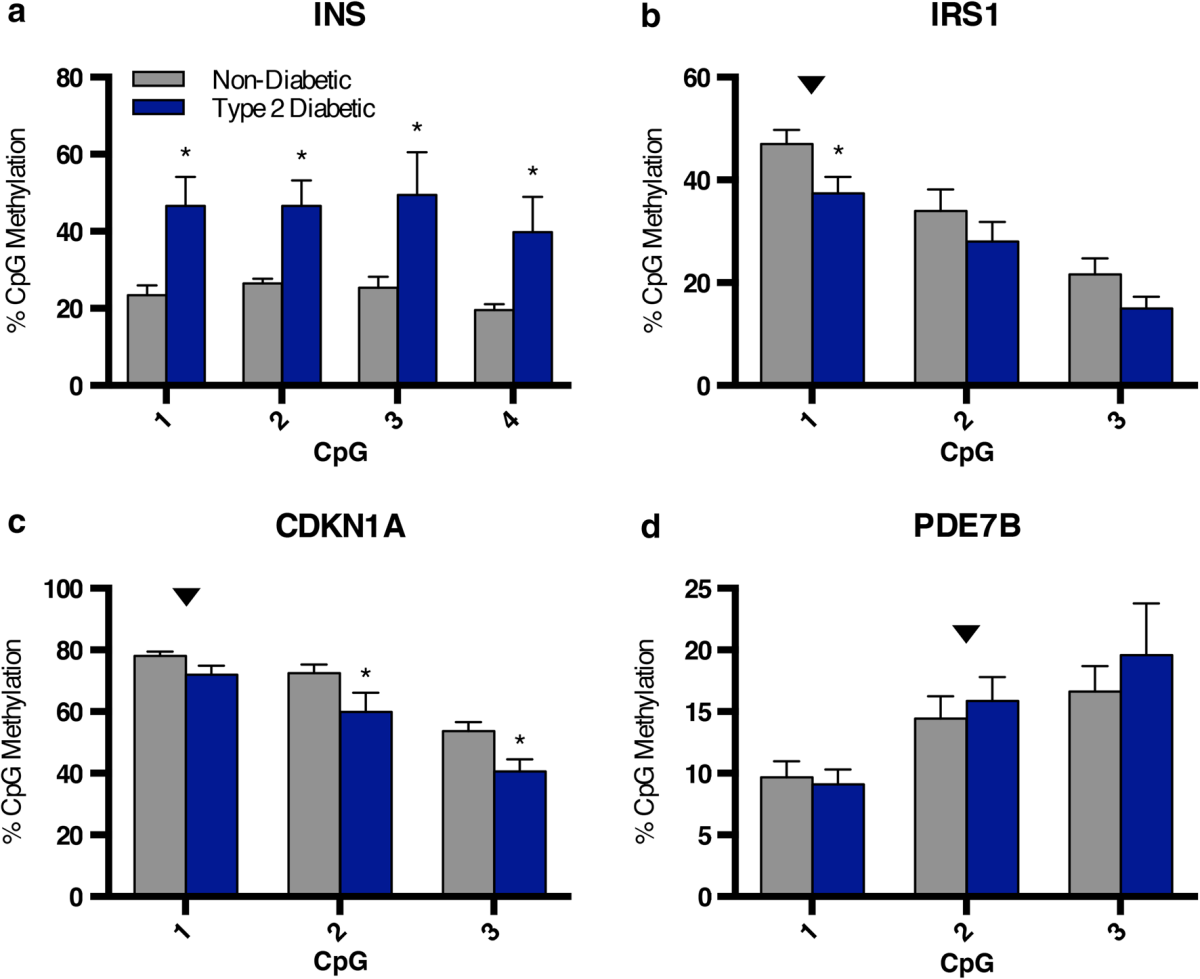
Validation of CpG loci differentially methylated in type 2 diabetic pancreatic islets by BisPCR^2^. Average percent CpG methylation in 5 non-diabetic and 5 type 2 diabetic human islet samples at loci previously shown to be differentially methylated in type 2 diabetic human islets, including **a** *INS*, **b** *IRS1*, **c** *CDKN1A*, and **d** *PDE7B*. *Black arrows* indicate CpGs analyzed previously by pyrosequencing by Dayeh and colleagues [18]. *p* value calculated by two-tailed *t* tests. *p < 0.05. *Error bars* indicate SEM.

We next sought to employ the BisPCR^2^ strategy to validate published differentially methylated loci in islets from type 2 diabetics [18]. We selected four genes, *INS*, *IRS1*, *CDKN1A*, and *PDE7B*, for validation. These loci were among those determined by Dayeh and colleagues to be differentially methylated in type 2 diabetic human islets in a genome-wide screen conducted with the Infinium HumanMethylation450 BeadChip array [18]. The insulin gene has also been described as differentially methylated in type 2 diabetic human islets through a candidate gene approach [19]. We designed PCR#1 primers targeting the region −112 to −336 base pairs upstream of the insulin transcription start site capturing four CpGs, three of which were previously reported to have increased DNA methylation in type 2 diabetic human islets [18]. We found all four CpGs measured had significantly increased DNA methylation (p < 0.05) with an average of 24% in non-diabetic compared to to 46% in type 2 diabetic samples (p < 0.0005) (Fig. 4a). This includes one CpG from the Infinium array that was not previously identified as differentially methylated, *INS* CpG #4 (cg24338752).

Individual CpGs within the other three loci had previously been re-analyzed by pyrosequencing, and we modified these pyrosequencing primers with PCR#1 adapter overhangs to adapt these amplicons to the BisPCR^2^ technology [18]. By using the BisPCR^2^ method with the same amplicons we were able to directly quantify DNA methylation at all CpGs within the target region. At the *IRS1* locus, we measured a comparable decrease of 10% CpG methylation in type 2 diabetic human islets, from 47 to 37%, at *IRS1* CpG #1 (cg04751089), as had been reported previously (Fig. 4b) [18]. In this amplicon we also determined DNA methylation at two adjacent CpGs that were not included in the Infinium array, and while the changes trended in a similar direction, there was no significant difference between non-diabetic and type 2 diabetic samples. At the *CDKN1A* locus, we did not find a significant difference in DNA methylation at the CpG previously analyzed by pyrosequencing, *CDKN1A* CpG #1 (cg21091547), but did observe a significant decrease of 10% at two adjacent CpGs captured in this amplicon (Fig. 4c). *CDKN1A* CpG #3 (cg24425727) was also identified as differentially methylated to a similar extent in the genome-wide study by Dayeh and colleagues, while *CDKN1A* CpG #2 (cg11920449) was not previously identified as differentially methylated [18]. Lastly, we did not observe a change in DNA methylation at any of the three CpGs assayed at the *PDE7B* locus (Fig. 4d). Thus, in our validation study using BisPCR^2^, we were able to confirm some previous genome-wide findings and not others, while making novel observations about additional nearby CpGs not covered in prior assays.

## Discussion

We were prompted to develop the BisPCR^2^ method by our need for a high-throughput, cost-effective method for interrogating multiple CpGs at base resolution within multiple target loci of interest. Fluorescence-based approaches to targeted bisulfite sequencing are limited by the number of CpGs that can be measured at one time, the inability to multiplex, and the reliance of measurements on a secondary enzymatic reaction. Next-generation sequencing techniques for targeted bisulfite sequencing employ the same strategy of bisulfite conversion and amplification of target loci, but result in a far more robust output by directly measuring base content of each CpG within an amplicon. Further, the ability to multiplex means that a single sequencing reaction can yield information about multiple target loci for multiple biological samples. One impediment of NGS approaches is the additional step of DNA sequencing library preparation following target enrichment, which can be expensive and time consuming. We have circumvented this challenge by modifying the target enrichment PCR primers with overhangs such that DNA sequencing libraries can be directly amplified from target enrichment amplicons. This modification dramatically decreases time and expense required for this NGS approach.

We technically validated the BisPCR^2^ method at the imprinted *MEG3* locus, which was previously shown to be hypermethylated in type 2 diabetic human islets [17]. We confirmed previous findings by both BisPCR^2^ and pyrosequencing and demonstrated that BisPCR^2^ measures DNA methylation at 19 CpGs, while pyrosequencing measures only two despite identical target sequence inputs. Further, our validation study of type 2 diabetes CpG risk loci highlights several important considerations about implementing targeted DNA methylation analysis, particularly as a diagnostic marker. In some instances, such as *IRS1*, our results were nearly identical to those reported by Dayeh and colleagues [18]. We found similar levels of DNA methylation in normal and type 2 diabetic human islets at *IRS* CpG #1 (cg04751089) and found no significant changes at two adjacent CpGs that were not probed for on the Infinium HumanMethylation450 BeadChip. However, in contrast to genome-wide findings, we did not observe a change in DNA methylation at *PDE7B* CpG #2 (cg27306443), or at adjacent CpGs. While this discrepancy may be due to our limited sample size, it stresses the point that CpG methylation at this locus is not a sufficient diagnostic indicator of type 2 diabetes. Our findings at the *CDKN1A* locus highlight a third point, as we did not measure a change in CpG methylation at the reported cg21091547 (*CDKN1A* CpG #1), but did find a significant decrease in methylation at two adjacent CpGs, one of which was also identified by Dayeh and colleagues [18]. These results again stress the danger of relying on a single CpG for reporting methylation changes, and also emphasize the value in incorporating multiple CpGs for the understanding of how DNA methylation is implicated in disease risk.

While we have demonstrated the utility of BisPCR^2^ in validating findings from genome-wide DNA methylation analyses, this technology is also suitable for other applications including candidate gene approaches and clinical diagnostic assays. In candidate gene approaches, where genome-wide analyses may not be possible, many regions can be surveyed simultaneously by pooling multiple PCR#1 products across larger sample sizes than would be feasible for fluorescence-based assays. Clinical applications can benefit as well by reducing the costs of NGS approaches while collecting high-resolution data about neighboring CpGs, the value of which was discussed earlier. It should also be noted that our particular study does not take full advantage of the sequencing capacity of the MiSeq, as 200,000 reads per amplicon is orders of magnitude beyond what would be sufficient to draw statistically significant conclusions. Considering a sequencing threshold of 1,000 reads per amplicon, the capacity of the number of amplicons and samples that can be analyzed in one run could be increased by two hundred-fold. This excess capacity could be distributed to additional samples or additional target loci, or a combination of both. We provide 48 single index barcoding primers based on widely used Illumina sequencing technology, which would accommodate 250 target loci per sample. Further, the barcoding primers could easily be modified with dual indices to increase multiplexing capacity, which may be of particular utility in a clinical assay.

## Conclusion

BisPCR^2^ is an efficient, cost-effective, and robust high-throughput technique for assessing DNA methylation at targeted loci of interest. Replacing DNA sequencing library preparation with a single round of PCR represents a significant improvement over other targeted bisulfite NGS approaches by reducing time and cost. This method is easily adaptable to different experimental setups to address a wide variety of biological questions relevant to DNA methylation.

## Methods

### Traditional targeted bisulfite NGS library preparation (BisPCRSeq)

100 ng of mouse genomic DNA, isolated from the intestinal epithelium of 3 month old C57BL/6 J-ApcMin/J mice (Jackson Laboratories), was bisulfite converted using the Epitect bisulfite kit (Qiagen). Template DNA was amplified using KAPA HIFI Uracel + (KAPA) with primers directed to the H19 locus (Forward: 5′-ATTAGTTAGTGTGGTTTATTATAGGAAG-3′ and Reverse: 5′-AACCATTCCAAAAATACACACATCTTA-3′). Sequencing libraries were made using the NEBNext Multiplex Sample Kit (NEB). These primers were also modified for incorporation into the BisPCR^2^ library preparation protocol, as described below.

### Genomic DNA isolation and sodium bisulfite conversion

Primary human islets were obtained from the Integrated Islet Distribution Program (IIDP). 10,000 islet equivalents were obtained from five non-diabetic and five type 2 diabetic donors. Genomic DNA (gDNA) was extracted using the Qiagen^®^ AllPrep DNA/RNA mini kit (Cat. No. 80204) following the manufacturer’s instructions. 500 ng of gDNA was treated with sodium bisulfite to convert unmethylated cytosines using the Qiagen^®^ EpiTect^®^ Bisulfite Kit (Cat. No. 59104). Reactions were carried out per the manufacturer’s protocol.

### Target enrichment (PCR#1)

Bisulfite-converted gDNA was PCR amplified to enrich for regions of interest for DNA methylation analysis. Primers directed to target regions were modified with the following partial adapter overhangs: PCR#1 Left Primer Overhang: 5′-ACACTCTTTCCCTACACGACGCTCTTCCGATCT-3′; PCR#1 Right Primer Overhang: 5′-GTGACTGGAGTTCAGACGTGTGCTCTTCCGATCT-3′. Primers directed to the *MEG3* and *INS* loci were designed using the Qiagen^®^ PyroMark assay Design software. Primers for *CDKN1A*, *PED7B* and *IRS1* were adapted from a recent study by Dayeh and colleagues [18]. Primer sequences and genomic coordinates are provided in Additional file 3: Table S1. PCR reactions were prepared with the Qiagen^®^ PyroMark PCR Kit (Cat. No. 978703) per the manufacturer’s recommendations using 2.8 ng of bisulfite-converted gDNA template per reaction and the suggested optimized cycling protocol. All PCR#1 products for individual biological samples were pooled based on relative band intensity when 5 μl of PCR#1 reaction from a representative sample, non-diabetic 1, was analyzed on a 1.5% agarose gel. Final amounts were combined as follows: *MEG3*: 6 μl, *CDKN1A*: 8 μl, *PED7B*: 4 μl, *IRS1*: 4 μl, *INS*: 4 μl, for a total of 26 μl per biological sample. Each pool of PCR products was purified with the Qiagen^®^ QIAquick PCR Purification Kit (Cat. No. 28104) per the manufacturer’s instructions.

### Sample barcoding (PCR#2)

Unique DNA sequencing barcodes were incorporated into each sample by a subsequent round of PCR amplification. Barcoding primers are provided in Additional file 4: Table S2. The Qiagen^®^ PyroMark PCR kit was used to amplify 1 ng of pooled PCR#1 template. Thermocycling conditions were modified to ensure consistent amplification of PCR products of different sizes and were as follows: 95°C—15 min; 10 cycles: 94°C—30 s, touchdown 68 to 56°C—30 s, 72°C—1 min; 72°C—10 min. PCR products were purified with Agencourt^®^ AMPure^®^ XP beads (Beckman Coulter, Cat. No. A63881). Sample concentrations were measured using the Qubit^®^ fluorometer (Life Technologies) dsDNA high sensitivity assay. Fragment length was determined by separating 1 ng of sample on an Agilent high sensitivity DNA assay using the 2100 Bioanalyzer (Agilent Technologies). The molarity of the libraries was quantified using the KAPA library quantification assay (Kapa Biosystems, Cat. No. KK4873).

### Next-generation sequencing

Next-generation sequencing was carried out on the Illumina MiSeq using Reagent Kit v2 following the manufacturer’s instructions. Briefly, a 2 nM pool of BisPCR^2^ libraries and 2 nM PhiX control were each denatured for 5 min with 0.2 N NaOH and diluted to final concentrations of 6 and 8 pM, respectively. The denatured pool was spiked with 10% denatured PhiX control and 600 μl of the prepared sample was loaded into the reagent cartridge. The sequencing reaction was carried out with 150 base pair paired-end sequencing. Sequences were aligned to an in silico bisulfite-converted human genome using the BS Seeker program, and any CpGs covered by the first sequencing read were ignored in the second sequencing read in paired-end sequencing [20]. The fraction of methylated cytosines was calculated as the merged frequency of cytosines for CpGs divided by total reads. Sequencing and DNA methylation analysis were carried out by the Next Generation Sequencing Core at the University of Pennsylvania (Philadelphia, PA, USA) [GEO: GSE69595].

### Pyrosequencing

Pyrosequencing was performed to technically validate BisPCR^2^ at the *MEG3* locus. Forward and reverse primers designed with Qiagen^®^ PyroMark Assay Design software were used for both methods, and for pyrosequencing the reverse primer was biotinylated. Pyrosequencing primer sequences were as follows: Forward: 5′-GGGGTGATAGTTTTTGGTTTATATT-3′, Reverse: 5′-CCATAACCAACACCCTATAAT-3′, Sequencing: 5′-TTTTTATATATTGTGTTTGAATTTA-3′. Bisulfite-converted genomic DNA from human islets, processed as described above, was amplified with the Qiagen^®^ PyroMark PCR Kit (Cat. No. 978703) per the manufacturer’s protocol. The pyrosequencing reaction was carried out using Qiagen^®^ PyroMark Gold Q96 CDT Reagents on the PyroMark Q96 MD (QIAGEN) according to the manufacturer’s instructions.

### Statistics

Data are shown as average ± SEM (*n* = 5). Average percent CpG methylation was compared by two-tailed *t* tests, and significance was defined as *p* < 0.05.

## Additional files

Additional file 1:
**Figure S1.** Diagram illustrating the design of BisPCR^2^ primer sequences. This diagram illustrates the details of adapter sequences that are introduced during PCR#1 and PCR#2. Purple and blue text indicates primer overhangs that are added to locus-specific forward and reverse primers, respectively, to amplify regions of interest in PCR#1. Target region is indicated by the series of “N’s” between adapters. PCR#2 primers, indicated by “F2″ and “R2″ forward and reverse primers, introduce the remainder of the adapter sequence as well as a unique index for each sample, shown in red. We have modified 48 different “R2,” or barcoding, primers, the sequences for which are provided in Supplemental Table 2. Additional file 2:
**Figure S2.** Comparison of BisPCR^2^ and traditional targeted bisulfite NGS methods. DNA methylation was measured in murine genomic DNA at the H19 locus using both BisPCR^2^ and traditional targeted bisulfite NGS (n = 3). Traditional targeted bisulfite NGS is denoted as BisPCRSeq. Additional file 3:
**Table S1.** PCR#1 primer sequences for amplification of bisulfite-converted genomic DNA. Forward and reverse target enrichment primers were modified with adapter overhangs. Locus-specific portion of primer sequences are in bold text and common adapter overhangs are in plain text. Additional file 4:
**Table S2.** PCR#2 primer sequences for library amplification and barcoding. Amplification with PCR#2 primers adds the remainder of adapter sequence and unique barcodes for up to 48 samples. A common forward primer, “Library_Primer1,” is used in combination with each unique barcoding reverse primer.

## Abbreviations

NGS: next-generation sequencing
GWAS: genome-wide association study
WGBS: whole genome bisulfite sequencing
PCR: polymerase chain reaction
gDNA: genomic DNA
ND: non-diabetic
T2D: type 2 diabetic
SEM: standard error of the mean

## References

[1] Callinan PA, Feinberg AP (2006). The emerging science of epigenomics. Hum Mol Genet.

[2] Reik W, Dean W, Walter J (2001). Epigenetic reprogramming in mammalian development. Science.

[3] Calvanese V, Lara E, Kahn A, Fraga MF (2009). The role of epigenetics in aging and age-related diseases. Ageing Res Rev.

[4] Sheaffer KL, Kim R, Aoki R, Elliott EN, Schug J, Burger L (2014). DNA methylation is required for the control of stem cell differentiation in the small intestine. Genes Dev.

[5] Lister R, Pelizzola M, Dowen RH, Hawkins RD, Hon G, Tonti-Filippini J (2009). Human DNA methylomes at base resolution show widespread epigenomic differences. Nature.

[6] Stadler MB, Murr R, Burger L, Ivanek R, Lienert F, Schoeler A (2011). DNA-binding factors shape the mouse methylome at distal regulatory regions. Nature.

[7] Ziller MJ, Gu H, Mueller F, Donaghey J, Tsai LT, Kohlbacher O (2013). Charting a dynamic DNA methylation landscape of the human genome. Nature.

[8] Baubec T, Schuebeler D (2014). Genomic patterns and context specific interpretation of DNA methylation. Curr Opin Genet Dev.

[9] Bergman Y, Cedar H (2013). DNA methylation dynamics in health and disease. Nat Struct Mol Biol.

[10] Heerboth S, Lapinska K, Snyder N, Leary M, Rollinson S, Sarkar S (2014). Use of epigenetic drugs in disease: an overview. Genet Epigenetics.

[11] Hamm CA, Costa FF (2015). Epigenomes as therapeutic targets. Pharmacol Ther.

[12] Petronis A (2010). Epigenetics as a unifying principle in the aetiology of complex traits and diseases. Nature.

[13] Rakyan VK, Down TA, Balding DJ, Beck S (2011). Epigenome-wide association studies for common human diseases. Nat Rev Genet.

[14] Lee E, Luo J, Wilson JM, Shi H (2013). Analyzing the cancer methylome through targeted bisulfite sequencing. Cancer Lett.

[15] Morrill BH, Cox L, Ward A, Heywood S, Prather RS, Isom SC (2013). Targeted DNA methylation analysis by high throughput sequencing in porcine peri-attachment embryos. J Reprod Dev.

[16] Masser DR, Berg AS, Freeman WM (2013). Focused, high accuracy 5-methylcytosine quantitation with base resolution by benchtop next-generation sequencing. Epigenetics Chromatin.

[17] Kameswaran V, Bramswig NC, McKenna LB, Penn M, Schug J, Hand NJ (2014). Epigenetic regulation of the DLK1-MEG3 MicroRNA cluster in human type 2 diabetic islets. Cell Metab.

[18] Dayeh T, Volkov P, Salo S, Hall E, Nilsson E, Olsson AH (2014). Genome-wide DNA methylation analysis of human pancreatic islets from type 2 diabetic and non-diabetic donors identifies candidate genes that influence insulin secretion. PLoS Genet.

[19] Yang BT, Dayeh TA, Kirkpatrick CL, Taneera J, Kumar R, Groop L (2011). Insulin promoter DNA methylation correlates negatively with insulin gene expression and positively with HbA(1c) levels in human pancreatic islets. Diabetologia.

[20] Chen P, Cokus SJ, Pellegrini M (2010). BS Seeker: precise mapping for bisulfite sequencing. BMC Bioinformatics.

