# Supplementary figures and images for: The BisPCR2 method for targeted bisulfite sequencing
Source: Epigenetics Chromatin. 2015 Aug 1;8:27. doi: 10.1186/s13072-015-0020-x (PMC4522100; doi:10.1186/s13072-015-0020-x)

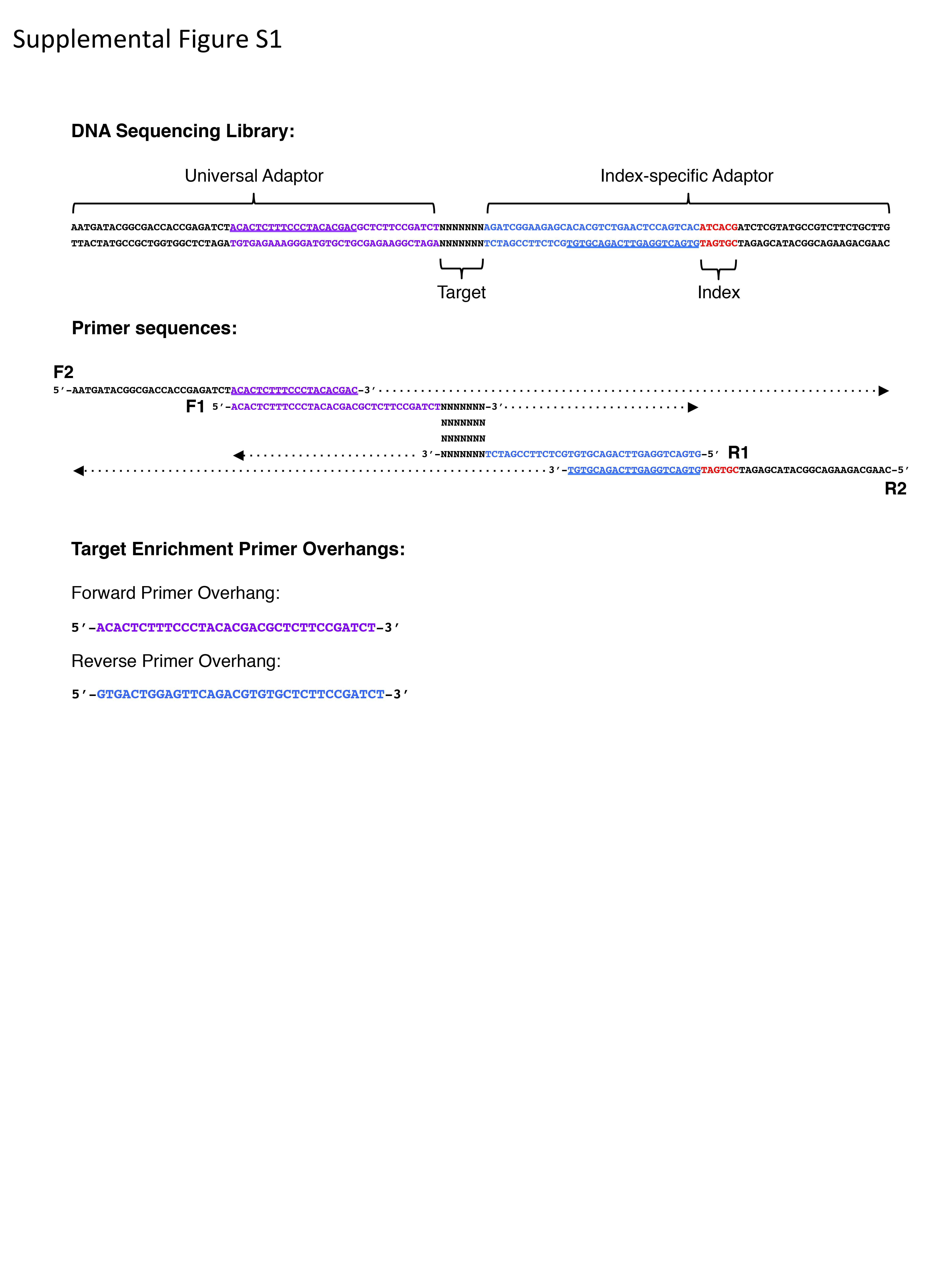

Supplement: Additional file 1: — Figure S1. Diagram illustrating the design of BisPCR2 primer sequences. This diagram illustrates the details of adapter sequences that are introduced during PCR#1 and PCR#2. Purple and blue text indicates primer overhangs that are added to locus-specific forward and reverse primers, respectively, to amplify regions of interest in PCR#1. Target region is indicated by the series of “N’s” between adapters. PCR#2 primers, indicated by “F2″ and “R2″ forward and reverse primers, introduce the remainder of the adapter sequence as well as a unique index for each sample, shown in red. We have modified 48 different “R2,” or barcoding, primers, the sequences for which are provided in Supplemental Table 2. [file 13072_2015_20_MOESM1_ESM.tif]

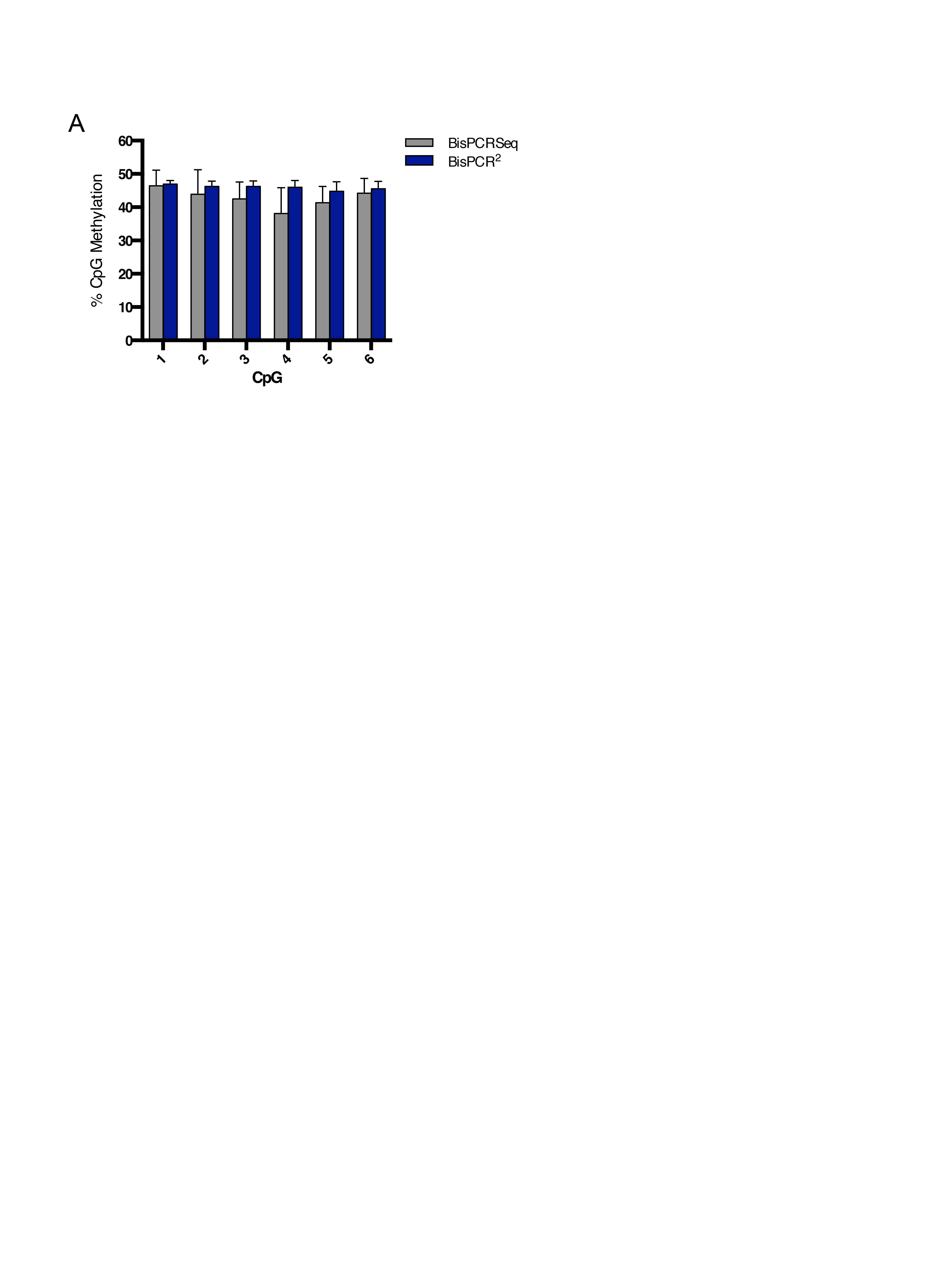

Supplement: Additional file 2: — Figure S2. Comparison of BisPCR2 and traditional targeted bisulfite NGS methods. DNA methylation was measured in murine genomic DNA at the H19 locus using both BisPCR2 and traditional targeted bisulfite NGS (n = 3). Traditional targeted bisulfite NGS is denoted as BisPCRSeq. [file 13072_2015_20_MOESM2_ESM.tif]
