# Supplementary material for: The BisPCR2 method for targeted bisulfite sequencing
Source: Epigenetics Chromatin. 2015 Aug 1;8:27. doi: 10.1186/s13072-015-0020-x (PMC4522100; doi:10.1186/s13072-015-0020-x)
Supplement: Additional file 3: — Table S1. PCR#1 primer sequences for amplification of bisulfite-converted genomic DNA. Forward and reverse target enrichment primers were modified with adapter overhangs. Locus-specific portion of primer sequences are in bold text and common adapter overhangs are in plain text. [file 13072_2015_20_MOESM3_ESM.docx]

**Supplemental Table 1.** PCR#1 primer sequences for amplification of bisulfite converted human genomic DNA

| **Primer** | **Sequence (5’ to 3’)** | **Coordinates** |
| --- | --- | --- |
| MEG3_F | ACACTCTTTCCCTACACGACGCTCT**T**CCGATCT**GGGGTGATAGTTTTTGGTTTATATT** | chr14:  101,291,952-101,292,257 |
| MEG3_R | GTGACTGGAGTTCAGACGTGTGCTCTTCCGATCT**CCATAACCAACACCCTATAAT** |  |
| CDKN1A_F | ACACTCTTTCCCTACACGACGCTCTTCCGATCT**GGAGTTATAGAAATAAAGGATGATAAGTAG** | chr6: 36,645,462-36,645,696 |
| CDKN1A_R | GTGACTGGAGTTCAGACGTGTGCTCTTCCGATCT**TCCCTATAATTACAACAACTTTATTAACCA** |  |
| PDE7B_F | ACACTCTTTCCCTACACGACGCTCTTCCGATCT**TGTTTTTTTTTGTTTGTGGTAATTGATAG** | chr6: 136,172,765-136,172,917 |
| PDE7B_R | GTGACTGGAGTTCAGACGTGTGCTCTTCCGATCT**TCCCTAAATAAATAACACCACTTTTCTC** |  |
| IRS1_F | ACACTCTTTCCCTACACGACGCTCTTCCGATCT**GGAAAGAATAGGAAGGGGTAG** | chr2: 227,659,611-227,659,781 |
| IRS1_R | GTGACTGGAGTTCAGACGTGTGCTCTTCCGATCT**ATTTAAACCCCTATACCAACATCAATTTCC** |  |
| INS_F | ACACTCTTTCCCTACACGACGCTCTTCCGATCT**TGGGGGTTGAGGTTGTAATT** | chr11: 2,182,551-2,182,775 |
| INS_R | GTGACTGGAGTTCAGACGTGTGCTCTTCCGATCT**ACCTCCAACTCTCCTAATCTAATAT** |  |
