# Supplementary material for: The BisPCR2 method for targeted bisulfite sequencing
Source: Epigenetics Chromatin. 2015 Aug 1;8:27. doi: 10.1186/s13072-015-0020-x (PMC4522100; doi:10.1186/s13072-015-0020-x)
Supplement: Additional file 4: — Table S2. PCR#2 primer sequences for library amplification and barcoding. Amplification with PCR#2 primers adds the remainder of adapter sequence and unique barcodes for up to 48 samples. A common forward primer, “Library_Primer1,” is used in combination with each unique barcoding reverse primer. [file 13072_2015_20_MOESM4_ESM.docx]

**Supplemental Table 2.** PCR#2 primer sequences for library amplification and barcoding.

| **Primer Name** | **Primer Sequence** | Barcode |
| --- | --- | --- |
| **Library_Primer1** | **AATGATACGGCGACCACCGAGATCTACACTCTTTCCCTACACGAC** | **ALL libraries** |
| Primer2_Index_1 | CAAGCAGAAGACGGCATACGAGAT**CGTGAT**GTGACTGGAGTTCAGACGTGT | ATCACG |
| Primer2_Index_2 | CAAGCAGAAGACGGCATACGAGAT**ACATCG**GTGACTGGAGTTCAGACGTGT | CGATGT |
| Primer2_Index_3 | CAAGCAGAAGACGGCATACGAGAT**GCCTAA**GTGACTGGAGTTCAGACGTGT | TTAGGC |
| Primer2_Index_4 | CAAGCAGAAGACGGCATACGAGAT**TGGTCA**GTGACTGGAGTTCAGACGTGT | TGACCA |
| Primer2_Index_5 | CAAGCAGAAGACGGCATACGAGAT**CACTGT**GTGACTGGAGTTCAGACGTGT | ACAGTG |
| Primer2_Index_6 | CAAGCAGAAGACGGCATACGAGAT**ATTGGC**GTGACTGGAGTTCAGACGTGT | GCCAAT |
| Primer2_Index_7 | CAAGCAGAAGACGGCATACGAGAT**GATCTG**GTGACTGGAGTTCAGACGTGT | CAGATC |
| Primer2_Index_8 | CAAGCAGAAGACGGCATACGAGAT**TCAAGT**GTGACTGGAGTTCAGACGTGT | ACTTGA |
| Primer2_Index_9 | CAAGCAGAAGACGGCATACGAGAT**CTGATC**GTGACTGGAGTTCAGACGTGT | GATCAG |
| Primer2_Index_10 | CAAGCAGAAGACGGCATACGAGAT**AAGCTA**GTGACTGGAGTTCAGACGTGT | TAGCTT |
| Primer2_Index_11 | CAAGCAGAAGACGGCATACGAGAT**GTAGCC**GTGACTGGAGTTCAGACGTGT | GGCTAC |
| Primer2_Index_12 | CAAGCAGAAGACGGCATACGAGAT**TACAAG**GTGACTGGAGTTCAGACGTGT | CTTGTA |
| Primer2_Index_13 | CAAGCAGAAGACGGCATACGAGAT**TTGACT**GTGACTGGAGTTCAGACGTGT | AGTCAA |
| Primer2_Index_14 | CAAGCAGAAGACGGCATACGAGAT**GGAACT**GTGACTGGAGTTCAGACGTGT | AGTTCC |
| Primer2_Index_15 | CAAGCAGAAGACGGCATACGAGAT**TGACAT**GTGACTGGAGTTCAGACGTGT | ATGTCA |
| Primer2_Index_16 | CAAGCAGAAGACGGCATACGAGAT**GGACGG**GTGACTGGAGTTCAGACGTGT | CCGTCC |
| Primer2_Index_17 | CAAGCAGAAGACGGCATACGAGAT**CTCTAC**GTGACTGGAGTTCAGACGTGT | GTAGAG |
| Primer2_Index_18 | CAAGCAGAAGACGGCATACGAGAT**GCGGAC**GTGACTGGAGTTCAGACGTGT | GTCCGC |
| Primer2_Index_19 | CAAGCAGAAGACGGCATACGAGAT**TTTCAC**GTGACTGGAGTTCAGACGTGT | GTGAAA |
| Primer2_Index_20 | CAAGCAGAAGACGGCATACGAGAT**GGCCAC**GTGACTGGAGTTCAGACGTGT | GTGGCC |
| Primer2_Index_21 | CAAGCAGAAGACGGCATACGAGAT**CGAAAC**GTGACTGGAGTTCAGACGTGT | GTTTCG |
| Primer2_Index_22 | CAAGCAGAAGACGGCATACGAGAT**CGTACG**GTGACTGGAGTTCAGACGTGT | CGTACG |
| Primer2_Index_23 | CAAGCAGAAGACGGCATACGAGAT**CCACTC**GTGACTGGAGTTCAGACGTGT | GAGTGG |
| Primer2_Index_24 | CAAGCAGAAGACGGCATACGAGAT**GCTACC**GTGACTGGAGTTCAGACGTGT | GGTAGC |
| Primer2_Index_25 | CAAGCAGAAGACGGCATACGAGAT**ATCAGT**GTGACTGGAGTTCAGACGTGT | ACTGAT |
| Primer2_Index_26 | CAAGCAGAAGACGGCATACGAGAT**GCTCAT**GTGACTGGAGTTCAGACGTGT | ATGAGC |
| Primer2_Index_27 | CAAGCAGAAGACGGCATACGAGAT**AGGAAT**GTGACTGGAGTTCAGACGTGT | ATTCCT |
| Primer2_Index_28 | CAAGCAGAAGACGGCATACGAGAT**CTTTTG**GTGACTGGAGTTCAGACGTGT | CAAAAG |
| Primer2_Index_29 | CAAGCAGAAGACGGCATACGAGAT**TAGTTG**GTGACTGGAGTTCAGACGTGT | CAACTA |
| Primer2_Index_30 | CAAGCAGAAGACGGCATACGAGAT**CCGGTG**GTGACTGGAGTTCAGACGTGT | CACCGG |
| Primer2_Index_31 | CAAGCAGAAGACGGCATACGAGAT**ATCGTG**GTGACTGGAGTTCAGACGTGT | CACGAT |
| Primer2_Index_32 | CAAGCAGAAGACGGCATACGAGAT**TGAGTG**GTGACTGGAGTTCAGACGTGT | CACTCA |
| Primer2_Index_33 | CAAGCAGAAGACGGCATACGAGAT**CGCCTG**GTGACTGGAGTTCAGACGTGT | CAGGCG |
| Primer2_Index_34 | CAAGCAGAAGACGGCATACGAGAT**GCCATG**GTGACTGGAGTTCAGACGTGT | CATGGC |
| Primer2_Index_35 | CAAGCAGAAGACGGCATACGAGAT**AAAATG**GTGACTGGAGTTCAGACGTGT | CATTTT |
| Primer2_Index_36 | CAAGCAGAAGACGGCATACGAGAT**TGTTGG**GTGACTGGAGTTCAGACGTGT | CCAACA |
| Primer2_Index_37 | CAAGCAGAAGACGGCATACGAGAT**ATTCCG**GTGACTGGAGTTCAGACGTGT | CGGAAT |
| Primer2_Index_38 | CAAGCAGAAGACGGCATACGAGAT**AGCTAG**GTGACTGGAGTTCAGACGTGT | CTAGCT |
| Primer2_Index_39 | CAAGCAGAAGACGGCATACGAGAT**GTATAG**GTGACTGGAGTTCAGACGTGT | CTATAC |
| Primer2_Index_40 | CAAGCAGAAGACGGCATACGAGAT**TCTGAG**GTGACTGGAGTTCAGACGTGT | CTCAGA |
| Primer2_Index_41 | CAAGCAGAAGACGGCATACGAGAT**GTCGTC**GTGACTGGAGTTCAGACGTGT | GACGAC |
| Primer2_Index_42 | CAAGCAGAAGACGGCATACGAGAT**CGATTA**GTGACTGGAGTTCAGACGTGT | TAATCG |
| Primer2_Index_43 | CAAGCAGAAGACGGCATACGAGAT**GCTGTA**GTGACTGGAGTTCAGACGTGT | TACAGC |
| Primer2_Index_44 | CAAGCAGAAGACGGCATACGAGAT**ATTATA**GTGACTGGAGTTCAGACGTGT | TATAAT |
| Primer2_Index_45 | CAAGCAGAAGACGGCATACGAGAT**GAATGA**GTGACTGGAGTTCAGACGTGT | TCATTC |
| Primer2_Index_46 | CAAGCAGAAGACGGCATACGAGAT**TCGGGA**GTGACTGGAGTTCAGACGTGT | TCCCGA |
| Primer2_Index_47 | CAAGCAGAAGACGGCATACGAGAT**CTTCGA**GTGACTGGAGTTCAGACGTGT | TCGAAG |
| Primer2_Index_48 | CAAGCAGAAGACGGCATACGAGAT**TGCCGA**GTGACTGGAGTTCAGACGTGT | TCGGCA |
